# Integration of Transcriptome and Metabolome Reveals the Genes and Metabolites Involved in *Bifidobacterium bifidum* Biofilm Formation

**DOI:** 10.3390/ijms22147596

**Published:** 2021-07-15

**Authors:** Zongmin Liu, Lingzhi Li, Zhifeng Fang, Yuankun Lee, Jianxin Zhao, Hao Zhang, Wei Chen, Haitao Li, Wenwei Lu

**Affiliations:** 1 State Key Laboratory of Food Science and Technology, Jiangnan University, Wuxi 214122, China; 7180112023@stu.jiangnan.edu.cn (Z.L.); lilingzhi@sina.cn (L.L.); zhifengf@foxmail.com (Z.F.); jxzhao@jiangnan.edu.cn (J.Z.); zhanghao@jiangnan.edu.cn (H.Z.); chenwei66@jiangnan.edu.cn (W.C.); liht@jiangnan.edu.cn (H.L.); 2 School of Food Science and Technology, Jiangnan University, Wuxi 214122, China; 3 Department of Microbiology & Immunology, Yong Loo Lin School of Medicine, National University of Singapore, Singapore 117545, Singapore; micleeyk@nus.edu.sg; 4 International Joint Research Laboratory for Pharmabiotics & Antibiotic Resistance, Jiangnan University, Wuxi 214122, China; 5 National Engineering Research Center for Functional Food, Jiangnan University, Wuxi 214122, China

**Keywords:** *Bifidobacterium bifidum*, biofilm, quorum sensing, two-component system, amino acid metabolism

## Abstract

*Bifidobacterium bifidum* strains, an important component of probiotic foods, can form biofilms on abiotic surfaces, leading to increased self-resistance. However, little is known about the molecular mechanism of *B. bifidum* biofilm formation. A time series transcriptome sequencing and untargeted metabolomics analysis of both *B. bifidum* biofilm and planktonic cells was performed to identify key genes and metabolites involved in biofilm formation. Two hundred thirty-five nonredundant differentially expressed genes (DEGs) (including *vanY*, *pstS*, *degP*, *groS*, *infC*, *groL*, *yajC*, *tadB* and *sigA*) and 219 nonredundant differentially expressed metabolites (including L-threonine, L-cystine, L-tyrosine, ascorbic acid, niacinamide, butyric acid and sphinganine) were identified. Thirteen pathways were identified during the integration of both transcriptomics and metabolomics data, including ABC transporters; quorum sensing; two-component system; oxidative phosphorylation; cysteine and methionine metabolism; glutathione metabolism; glycine, serine and threonine metabolism; and valine, leucine and isoleucine biosynthesis. The DEGs that relate to the integration pathways included *asd*, *atpB*, *degP*, *folC*, *ilvE*, *metC*, *pheA*, *pstS*, *pyrE*, *serB*, *ulaE*, *yajC* and *zwf*. The differentially accumulated metabolites included L-cystine, L-serine, L-threonine, L-tyrosine, methylmalonate, monodehydroascorbate, nicotinamide, orthophosphate, spermine and tocopherol. These results indicate that quorum sensing, two-component system and amino acid metabolism are essential during *B. bifidum* biofilm formation.

## 1. Introduction

Biofilm is the aggregation of microorganisms in which cells are frequently embedded in a self-produced matrix of extracellular polymeric substances (EPSs) that are adherent to each other and/or a surface [1]. These EPSs are mainly polysaccharides, proteins, lipids and nucleic acids that provide mechanical stability of biofilms and form a three-dimensional polymer network that interconnects and immobilizes biofilm cells [2]. A bacterial biofilm is an emergent form of life based on an EPS matrix representing a protected mode for bacterial cells, which exhibit a set of characteristic features such as desiccation tolerance, resource capture, social cooperation and antimicrobial resistance [3]. Biofilm formation is a complex process involving multiple steps, including initial attachment, accumulation, maturation and dispersion [4].

Bifidobacteria, particularly *Bifidobacterium bifidum*, are believed to be the first colonizers of the human gastrointestinal tract; they can form biofilms on mucosa and food residues in the gut lumen [5]. The ability of various strains of *Bifidobacterium* to form biofilms on abiotic surfaces, such as glass, stainless steel, polystyrene and complex food matrices, has been well documented [6,7,8]. Environmental stresses, including oxidative stress, bile, low pH value and nutrient starvation, can induce bacterial biofilm formation [9]. Oxygen exposure causes changes in fatty acids in the bifidobacteria cells and an extension of the lag phase of growth; the cells become elongated and develop a rough surface with many nodes due to abnormal or incomplete cell division, and the oxygen-tolerant bifidobacteria seem to defend against oxygen stress through the increase in the content of short fatty acids and cyclopropane fatty acids and the induction of an oxygen stress protein [10]. Transcriptomic analysis of *Bifidobacterium longum* subsp. *longum* BBMN68 in response to oxidative shock reveals that 3% oxygen treatment can cause oxidative stress, which contributes to biofilm formation [11]. High concentrations of bile (0.5% and above) may lyse bifidobacterial cells, leading to the release of intracellular signals such as oligopeptides or autoinducer-2 (AI-2) to trigger quorum sensing; extracellular DNA released from lysed cells may also coat the surface and result in additional electrostatic interactions, allowing the adherence of bifidobacteria [12].

Metabolomic analysis has been widely applied to study bacterial biofilm, providing information on metabolic state during biofilm formation [13,14]. The metabolomic results of *Helicobacter pylori* presented as two distinctly different groups: low-biofilm-formers produced more metabolites than high-biofilm-formers [13]. Liquid chromatography coupled with mass spectrometry (LC-MS) analysis reveals biological and metabolic processes essential for *Candida albicans* biofilm growth [15]. Untargeted metabolomics revealed that the metabolic state of *B. bifidum* in the biofilm and planktonic states were different, mainly in aminoacyl-tRNA biosynthesis; alanine, aspartate and glutamate metabolism; arginine and proline metabolism; citrate cycle and nitrogen metabolism; and exopolysaccharide biosynthesis (serves as a major component of biofilm matrix) [16]. The beneficial effects of probiotic biofilms, especially those of *Bifidobacterium* and *Lactobacillus*, have gained special interest from academia and the food industry [7,8,17].

The aim of this study was to identify the key genes and metabolites involved in *B. bifidum* biofilm formation. Here, we established a bifidobacterial biofilm fermentation system that contains wheat fiber (WF) as a carrier and combined a time series transcriptome sequencing and untargeted metabolomics analysis of both biofilm and planktonic cells. Field-emission scanning electron microscopy (FESEM) was used to observe the biofilm cell morphology and extracellular matrix. Our analysis provides insights into the mechanism of bifidobacterial biofilm formation.

## 2. Results

### 2.1. B. bifidum FHB150 Form Biofilm on WF

**Figure 1:**
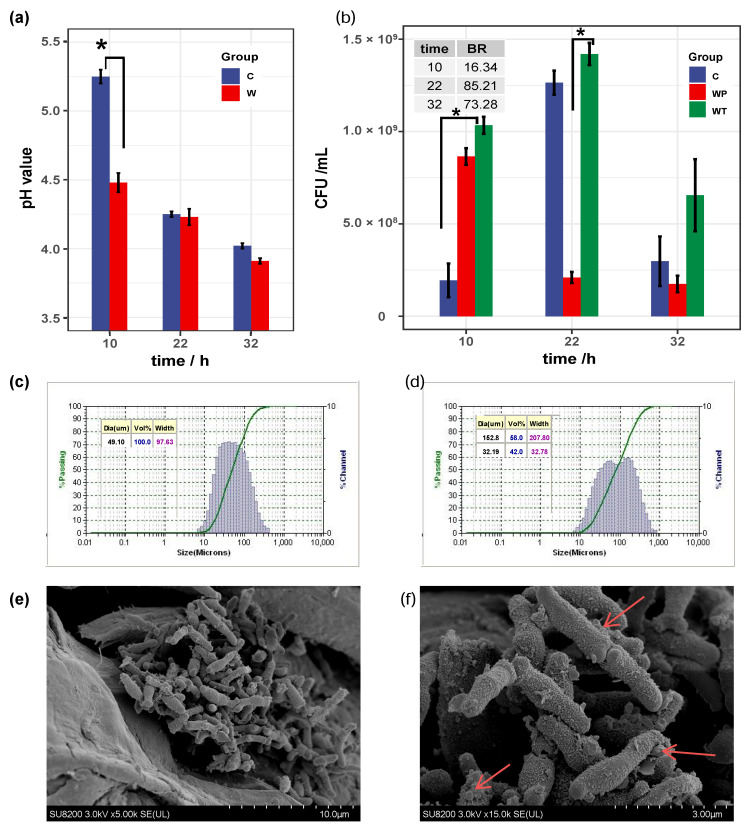
*B. bifidum* FHB150 form biofilm on WF. WF: wheat fiber. (**a**) pH values of WF culture and control culture. Significance is expressed in comparison with the controls at the same time (* *p* < 0.05 are significantly different). (**b**) Cell number of WF culture and control culture. WT: Total cell number in WF culture. WP: Planktonic cell number in EF culture; C: Cell number in control culture; BR: biofilm rate; BR = (WT − WP)/WT * 100%. (**c**) Particle size of noninoculated WF. (**d**) Particle size of inoculated WF at 22 h. (**e**) FESEM of biofilm on WF at 22 h, 5000×. (**f**) FESEM of biofilm on WF at 22 h, 15,000×; the presence of EPSs is indicated by red arrows. EPSs: extracellular polymeric substances.

In this study, we used WF as a carrier in the fermentation system and evaluated the biofilm formation by the biofilm formation rate, carrier particle size and FESEM. The pH values in the control and WF cultures of *B. bifidum* were decreased during fermentation and lower than 4.0 at 32 h (Figure 1a). The pH value in WF culture was significantly lower than that in control culture at 10 h (*p* < 0.05). There were 1.95 × 10^8^ and 1.03 × 10^9^ CFU/mL in the control culture and the WF culture at 10 h (Figure 1b), respectively (*p* < 0.05). These results suggest that cells attached to carriers in the early fermentation stage and proliferated rapidly, producing more acidic substances than control culture. Figure 1c shows WF with an average particle size of around 50 µm. However, the average particle size was over 150 µm (Figure 1d) with the biofilm rate higher than 85% (Figure 1b) at 22 h. The FESEM results showed that *B. bifidum* cells adhered to the surface of WF (Figure 1e) and secreted extracellular substances to form the biofilm (Figure 1f). The biofilm rate, carrier particle size and FESEM results indicate that the formation of *B. bifidum* biofilm includes the adsorption of cells to the carrier at the initial stage (0–10 h), the growth and development of the biofilm (22–32 h) and the dispersion of the biofilm.

### 2.2. Key Genes Involved in B. bifidum Biofilm Formation

#### 2.2.1. Two Hundred Thirty-Five Nonredundant DEGs during the Biofilm Formation Were Identified

**Figure 2:**
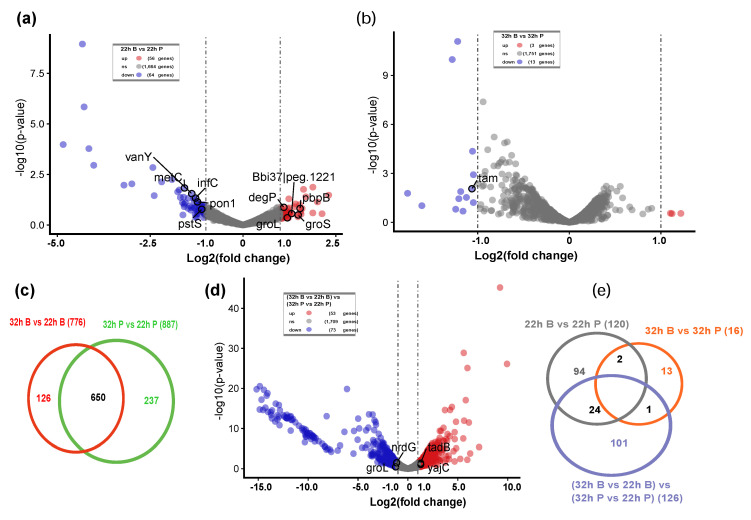
Transcript profiling changes during *B. bifidum* FHB150 biofilm formation. (**a**) The volcano plot showing the DEGs between 22 h B and 22 h P. DEGs: differentially expressed genes. Red points represent upregulated DEGs and blue points represent downregulated DEGs. B: biofilm cells, the group containing wheat fiber. P: planktonic cells, the control group without wheat fiber. (**b**) The volcano plot showing the DEGs between 32 h B and 32 h P. (**c**) Venn diagram representing the number of DEGs between 32 h B vs. 22 h B and 32 h P vs. 22 h P. (**d**) The volcano plot showing the DEGs changes during biofilm formation (22–32 h). (**e**) Venn diagram representing the number of nonredundant DEGs during biofilm formation.

Biofilm formation is a complex dynamic process, generally established through surface attachment, biofilm maturation and biofilm dispersion [18]. To identify the key differentially expressed genes (DEGs) during *B. bifidum* biofilm formation, transcriptomic analysis was conducted on both biofilm and planktonic samples collected at 22 and 32 h. Specified pairwise transcriptome comparisons were performed: 22 h B vs. 22 h P; 32 h B vs. 32 h P; 32 h B vs. 22 h B; 32 h P vs. 22 h P. There were 120 DEGs in 22 h B vs. 22 h P (56 DEGs upregulated, 64 downregulated) (Figure 2a). However, there were only 16 DEGs in 32 h B vs. 32 h P (3 upregulated, 13 downregulated) (Figure 2b), indicating that there was little difference in gene expression between the biofilm and planktonic cell state. To further explore the DEGs related to biofilm growth from 22 to 32 h, we compared the cell growth gene changes in WF group (32 h B vs. 22 h B) and control group (32 h P vs. 22 h P) (Figure 2c). This analysis yielded 126 DEGs associated with biofilm growth, including 53 upregulated and 73 downregulated genes (Figure 2d). The Venn diagram shows the 235 nonredundant DEGs during *B. bifidum* biofilm formation, including *vanY*, *pstS*, *degP*, *groS*, *infC*, *groL*, *yajC* and *tadB* (Figure 2e).

#### 2.2.2. Function of DEGs during *B. bifidum* Biofilm Formation

**Figure 3:**
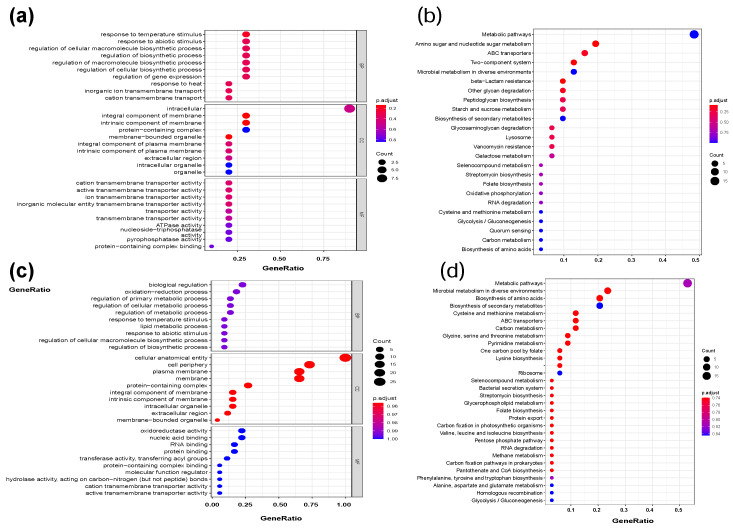
Function analysis of nonredundant DEGs during *B. bifidum* FHB150 biofilm formation. The GO terms analysis (**a**) and KEGG analysis (**b**) of DEGs between 22 h WF and 22 h C. GO: Gene Ontology. KEGG: Kyoto Encyclopedia of Genes and Genomes.The GO terms analysis (**c**) and KEGG analysis (**d**) of DEGs between 32 h B vs. 22 h B and 32 h P vs. 22 h P.

To investigate the function of these nonredundant DEGs during *B. bifidum* biofilm formation, we performed a functional analysis using Gene Ontology (GO) and Kyoto Encyclopedia of Genes and Genomes (KEGG) pathway terms (Figure 3). Thirteen genes (*budA*, *cscA*, *folC*, *groL*, *groS*, *infC*, *mgtA*, *mrp*, *nrdG*, *rarD*, *tam*, *ulaE*) were categorized into “biological process” (BP), “cellular component” (CC) and “molecular function” (CF) GO terms, including “response to abiotic stimulus”, “regulation of gene expression”, “extracellular region”, “transporter activity” and “peptide biosynthetic process”, and the top 10 terms are shown in Figure 3a. Meanwhile, Figure 3b shows the KEGG pathways of DEGs, including “two-component system” (Bbi37|peg.1341, *degP*, *pstS*, *vanY*), “amino sugar and nucleotide sugar metabolism” (Bbi37|peg.282, Bbi37|peg.249, Bbi37|peg.376, Bbi37|peg.680, Bbi37|peg.1418, Bbi37|peg.1494), “peptidoglycan biosynthesis” (*pbpB*, *pon1*, *vanY*), “quorum sensing” (Bbi37|peg.1221) and “biosynthesis of amino acids” (*metC*). There was only one functional DEG (*tam*) identified in 32 h that was labeled “interspecies interaction between organisms”. Interestingly, the DEGs from 22 to 32 h were associated with functional terms, including “response to stress” (*infC*, *groL*), “oxidation–reduction process” (*asd*, *bcp*, *nrdG*), “cysteine and methionine metabolism” (*asd*, *cysB*, *ilvE*, *metC*), “lysine biosynthesis” (*asd*, *dapD*), “alanine, aspartate and glutamate metabolism” (*iaaA*), “lipid metabolic process” (*pldB*, *dus*) and “bacterial secretion system, quorum sensing” (*yajC*) (Figure 3c,d). The function of DEGs indicates that biofilm formation of *B. bifidum* was related to abiotic stimulation, stress response, protein and polysaccharide biosynthesis, two-component system, quorum sensing and global regulatory factors.

**Figure 4:**
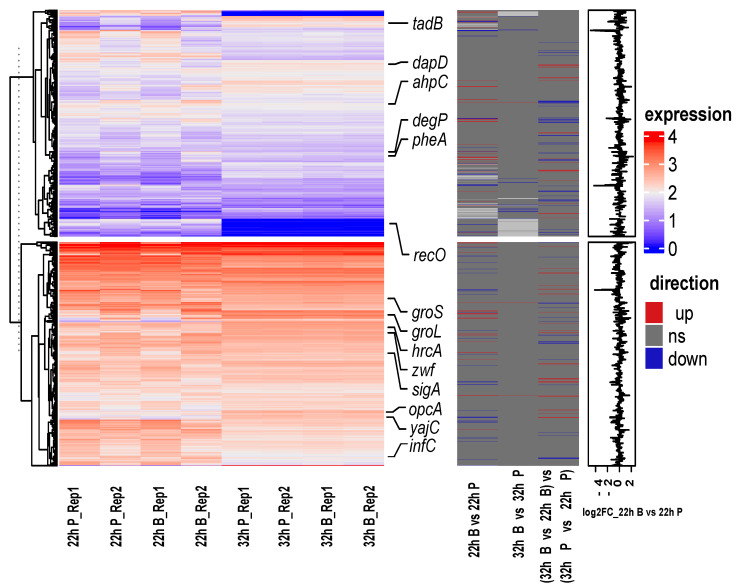
Gene expression heatmap of *B. bifidum* FHB150. The left heat map shows the gene expression levels in different samples. Red indicates a higher gene expression level, and blue indicates a lower gene expression level. The color from red to blue indicates log10 (FPKM +1) from high to low. The middle heat map shows the three directions of genes, upregulated, downregulated or ns (no significance). FPKM: fragments per kilobase of exon per million mapped fragments. The right bar graph is the differential expression log2 FC of genes in 22 h B vs. 22 h P. FC: fold change.

Genes with similar expression patterns may have similar functions, which makes it is possible to predict DEGs’ function without annotation names involved in *B. bifidum* biofilm formation. The fragments per kilobase of exon per million mapped fragments (FPKM) was used to conduct the hierarchical clustering analysis of transcript abundance, and 1890 genes were divided into two clusters (high and low) based on the expression of genes (Figure 4). Remarkably, 22 h B vs. 22 h P were involved in 120 DEGs, of which 25 DEGs were shared with 32 h B vs. 22 h B. There were 10 DEGs (*metC*, *infC*, *ybjQ*, Bbi37|rna.25, Bbi37|peg.509, Bbi37|peg.510, Bbi37|peg.650, Bbi37|peg.1181 and Bbi37|peg.1182 were highly expressed, while *gla* was lowly expressed) downregulated in 22 h B vs. 22 h P but upregulated in 32 h B vs. 22 h B. Fifteen DEGs (*groL*, *gyrB2*, *mug*, *nrdG*, *ydeD* and *Bbi37|peg.1148* were highly expressed, while *rarD*, *Bbi37|rna.24*, *Bbi37|peg.1148*, *Bbi37|peg.227*, *Bbi37|peg.965*, *Bbi37|peg.1250*, *Bbi37|peg.1336*, *Bbi37|peg.1728* and *Bbi37|peg.1733* were lowly expressed) were upregulated in 22 h B vs. 22 h P but downregulated in 32 h B vs. 22 h B.

#### 2.2.3. The Interaction of DEGs during *B. bifidum* Biofilm Formation

**Figure 5:**
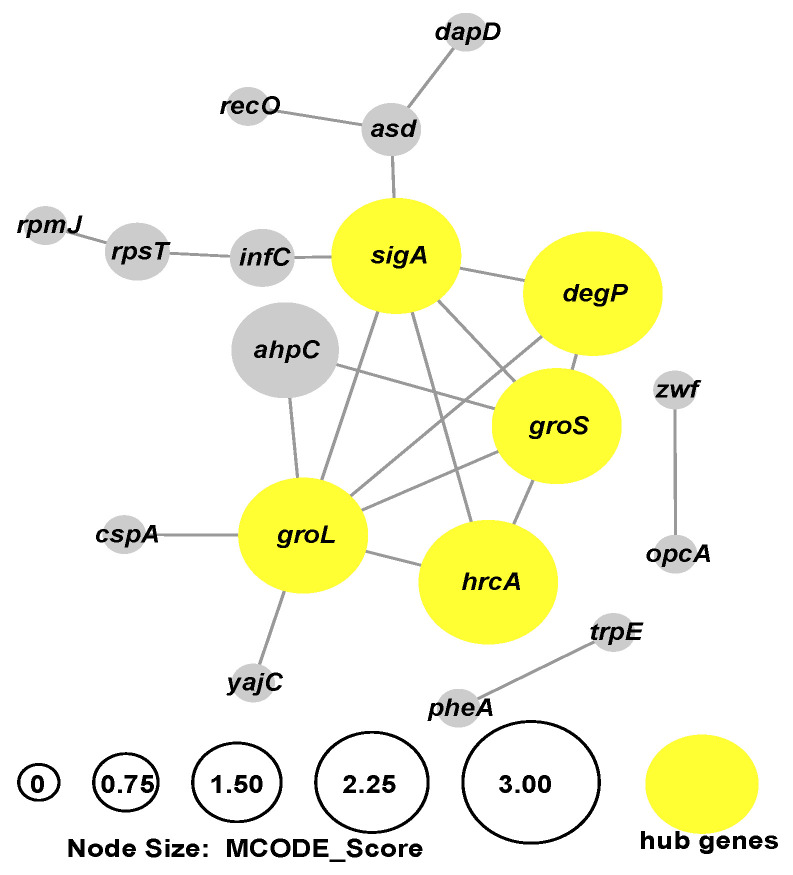
*B. bifidum* biofilm formation PPI network. PPI: protein–protein interaction. The node size was determined by MCODE degree. Yellow nodes present the hub genes.

To further investigate the interaction of these nonredundant DEGs during *B. bifidum* biofilm formation, we used the STRING database to identify potential interactions between them. A protein–protein interaction (PPI) network between DEGs was constructed, and five hub genes (*degP*, *groL*, *groS*, *hrcA*, *sigA*) were identified by MCODE (Figure 5). Despite there being 235 nonredundant genes that were differentially expressed, the final PPI network only had 18 genes, which means that the interaction of most DEGs during biofilm formation is still unknown. *degP* was related to two-component system, upregulated during biofilm formation (22 h B vs. 22 h P, 32 h B vs. 22 h B). *groL* (prevents misfolding and promotes the refolding and proper assembly of unfolded polypeptides generated under stress conditions) and *groS* (binds to Cpn60 in the presence of Mg-ATP and suppresses the ATPase activity of the latter) were both upregulated in 22 h B vs. 22 h P, related to the stress response. *sigA* (the primary sigma factor during exponential growth) was also upregulated in 22 h B vs. 22 h P. *hrcA*, negative regulator of *groELS* operons, was downregulated in 32 h B vs. 22 h B. Notably, *yajC* (preprotein translocase subunit) associated with protein export and quorum sensing was 2.44-fold upregulated in 32 h B vs. 22 h B. The interaction of DEGs indicates that two-component system, quorum sensing and protein biosynthesis were upregulated after *B. bifidum* was stimulated by stress during biofilm growth.

### 2.3. Main Metabolites Involved in B. bifidum Biofilm Formation

#### 2.3.1. Metabolite Profiling Changes and Enrichment Pathway Analysis during Biofilm Formation

**Figure 6:**
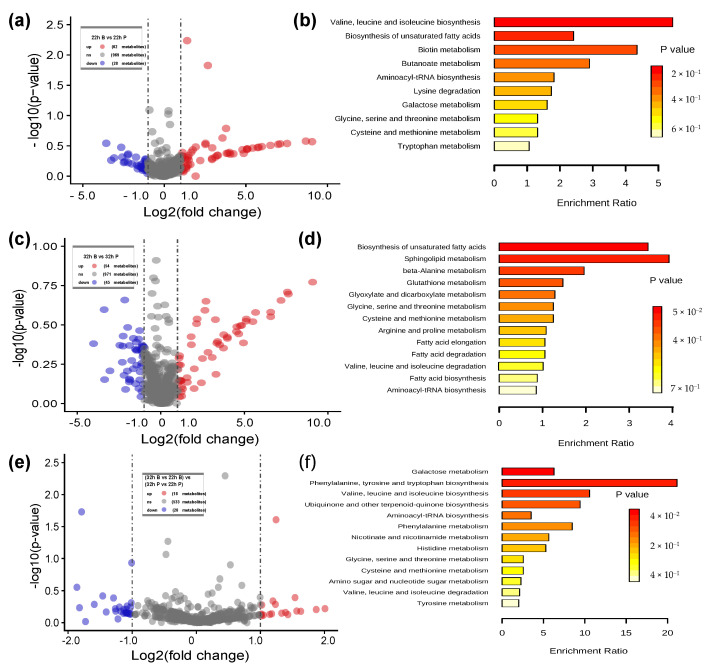
Metabolite profiling changes and enrichment pathway analysis during biofilm formation. The volcano plot showing the upregulated (red points) and downregulated (blue points) metabolites (**a**) and enrichment pathway analysis of these metabolites (**b**) between 22 h B and 22 h P. The volcano plot showing the upregulated and downregulated metabolites (**c**) and enrichment pathway analysis of these metabolites (**d**) between 32 h B and 32 h P. The volcano plot showing the upregulated and downregulated metabolites (**e**) and enrichment pathway analysis of these metabolites (**f**) between 32 h B vs. 22 h B and 32 h P vs. 22 h P (22–32 h was the biofilm growth period).

LC-MS data analysis of biofilm (cells on the WF surface) and planktonic (cells in control group) metabolites yielded 1070 molecular features in total (Figure 6). There were 62 metabolites upregulated in 22 h B vs. 22 h P with FC of ≥2 and 39 metabolites downregulated with FC ≤ −2 (a). Figure 6b shows that the enriched pathways of these differentially expressed metabolites (arachidic acid, butyric acid, eicosapentaenoic acid, L-cystine, L-lysine, L-threonine, melatonin) included valine, leucine and isoleucine biosynthesis; biosynthesis of unsaturated fatty acids; biotin metabolism; butanoate metabolism; aminoacyl-tRNA biosynthesis; lysine degradation; galactose metabolism; glycine, serine and threonine metabolism; cysteine and methionine metabolism; and tryptophan metabolism. 

In 32 h WF vs. 32 h C, 54 metabolites were upregulated and 45 metabolites were downregulated (Figure 6c). There were seven metabolites (palmitic acid, arachidic acid, L-serine, methylmalonic acid, palmitic acid, spermine, sphinganine) in 32 h B vs. 32 h P (Figure 6d) associated with biosynthesis of unsaturated fatty acids; sphingolipid metabolism; beta-alanine metabolism; glutathione metabolism; glyoxylate and dicarboxylate metabolism; glycine, serine and threonine metabolism; cysteine and methionine metabolism; arginine and proline metabolism; valine, leucine and isoleucine degradation; and aminoacyl-tRNA biosynthesis.

Only 46 metabolites showed 2 FC (18 metabolites upregulated, 28 metabolites downregulated) between 32 h B vs. 22 h B and 32 h P vs. 22 h P (22–32 h was the biofilm growth period) (Figure 6e). However, these differentially expressed metabolites associated with the most enriched pathway (D-galactose, histamine, L-cystine, L-threonine, L-tyrosine, methylmalonic acid, niacinamide): galactose metabolism; phenylalanine, tyrosine and tryptophan biosynthesis; valine, leucine and isoleucine biosynthesis; ubiquinone and other terpenoid–quinone biosynthesis; aminoacyl-tRNA biosynthesis; phenylalanine metabolism; nicotinate and nicotinamide metabolism; histidine metabolism; glycine, serine and threonine metabolism; cysteine and methionine metabolism; amino sugar and nucleotide sugar metabolism; valine, leucine and isoleucine degradation; and tyrosine metabolism (Figure 6f).

#### 2.3.2. Function of Differentially Expressed Metabolites

**Figure 7:**
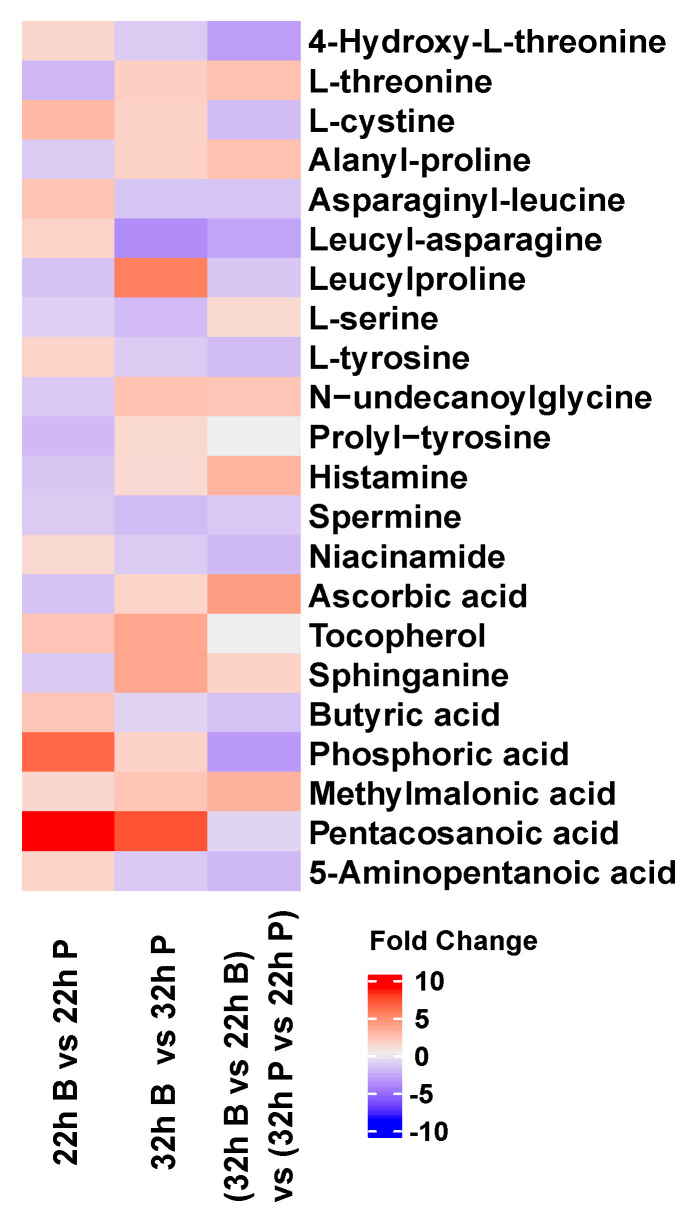
Heatmap of key metabolites during biofilm formation. Red represents upregulated and blue represents downregulated.

To further study the key metabolites during biofilm formation, we used MetaboAnalyst 5.0 to search the function of 219 nonredundant differentially expressed metabolites. Notably, 22 metabolites were mainly amino acids, short-chain fatty acids and vitamins (Figure 7). L-Cystine (KEGG C00491), asparaginyl-leucine (HMP HMDB0028735), tocopherol (KEGG C02483), butyric acid (KEGG C00246), phosphoric acid (KEGG C00009) and pentacosanoic acid (HMP HMDB0002361) were respectively upregulated 2.67-, 2.11-, 2.10-, 2.03-, 6.37-, and 8.70-fold in 22 h B vs. 22 h P. Leucylproline (HMP HMDB0011175), N-undecanoylglycine (HMP HMDB0013286), tocopherol, sphinganine (KEGG C00836), methylmalonic acid (KEGG C02170) and pentacosanoic acid were respectively upregulated 5.39-, 2.34-, 3.52-, 3.55-, 2.08- and 7.10-fold in 32 h B vs. 32 h P. Leucyl-asparagine (HMP HMDB0028924), L-serine (KEGG C00065) and spermine (KEGG C00750) were respectively downregulated 3.95-, 2.08- and 2.14-fold in 32 h B vs. 32 h P. L-Threonine, alanyl-proline (HMP HMDB0028695), N-undecanoylglycine, histamine (KEGG C00388), ascorbic acid (KEGG C01041) and methylmalonic acid were upregulated, while 4-hydroxy-L-threonine, L-cystine, leucyl-asparagine, L-tyrosine (KEGG C00082), niacinamide (KEGG C00153), phosphoric acid and 5-aminopentanoic acid (KEGG C00431) were downregulated during biofilm growth (32 h B vs. 22 h B) vs. (32 h P vs. 22 h P). This finding indicates that amino acids, short-chain fatty acids and vitamins were key metabolites and their expression levels were different during biofilm formation.

### 2.4. Thirteen Pathways Were Identified during the Integration of Both Transcriptomics and Metabolomics Data

**Figure 8:**
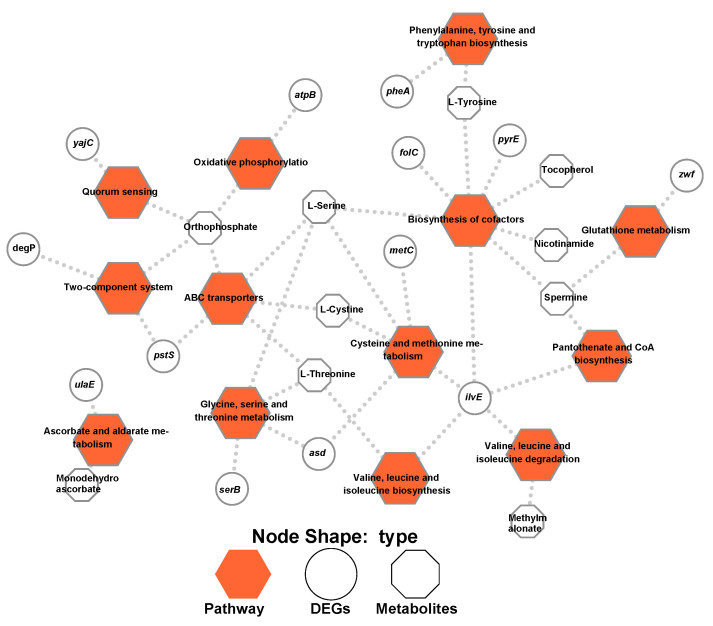
Integration of transcriptomic and metabolomic data. Hexagon, ellipse and octagon nodes indicate KEGG pathways, DEGs and differently expressed metabolites, respectively.

To provide a more comprehensive understanding of biofilm formation, the transcript–metabolite interaction network was generated for DEGs and the differentially expressed metabolites. The gene–metabolite interaction consists of 36 nodes connected via 41 edges (Figure 8). Thirteen pathways were identified during the integration of both transcriptomics and metabolomics data, including ABC transporters; ascorbate and aldarate metabolism; biosynthesis of cofactors; cysteine and methionine metabolism; glutathione metabolism; glycine, serine and threonine metabolism; oxidative phosphorylation; pantothenate and CoA biosynthesis; phenylalanine, tyrosine and tryptophan biosynthesis; quorum sensing; two-component system; valine, leucine and isoleucine biosynthesis; and valine, leucine and isoleucine degradation. The DEGs that relate to the integration pathways included *asd*, *atpB*, *degP*, *folC*, *ilvE*, *metC*, *pheA*, *pstS*, *pyrE*, *serB*, *ulaE*, *yajC* and *zwf*. The differentially accumulated metabolites included L-cystine, L-serine, L-threonine, L-tyrosine, methylmalonate, monodehydroascorbate, nicotinamide, orthophosphate, spermine and tocopherol. Overall, the integration analysis successfully identified pathways that may be associated with biofilm formation.

## 3. Discussion

In this study, we firstly collected samples from *B. bifidum* under biofilm (cells on the WF surface) and planktonic (cells in the control group) conditions during fermentation and then conducted a combined transcriptomic and metabolomic analysis to determine the key genes and metabolites involved in *B. bifidum* biofilm formation. Two hundred thirty-five nonredundant DEGs (including *vanY*, *pstS*, *degP*, *groS*, *infC*, *groL*, *yajC*, *tadB* and *sigA*) and 219 nonredundant differentially expressed metabolites (including L-threonine, L-cystine, L-tyrosine, ascorbic acid, niacinamide, butyric acid and sphinganine) were identified during the biofilm formation. Thirteen pathways were identified during the integration of both transcriptomics and metabolomics data, including ABC transporters; quorum sensing; two-component system; oxidative phosphorylation; ascorbate and aldarate metabolism; cysteine and methionine metabolism; glutathione metabolism; glycine, serine and threonine metabolism; and valine, leucine and isoleucine biosynthesis. These results indicate that quorum sensing, two-component system and amino acid metabolism are essential during *B. bifidum* biofilm formation.

Various methods that rely on molecular and microbiological methods or on chemical or physical properties of the biofilm can be used to assess biofilm biomass and viability [19]. Microscopy methods are important tools for assessing biofilm biomass properties in a more direct way, allowing the description of biofilm heterogeneities, spatial organization and links with the community functions [20,21]. In addition, the diameter change of the biofilm in a dynamic fermentation system can reflect the growth rate of the biofilm [22]. Here, we used WF (with an average particle size of around 50 µm) as a carrier in the fermentation system and evaluated the biofilm formation by the biofilm formation rate, carrier particle size and FESEM. The average biofilm particle size was over 150 µm with the biofilm rate higher than 85% at 22 h (Figure 1). The number of viable bacteria on the WF indicates that the formation of *B. bifidum* biofilm includes the adsorption of cells to the carrier at the initial stage, the growth and development of the biofilm (22–32 h) and the dispersion of the biofilm. Carriers with porous structures, such as grape seeds, may contribute to biofilm formation on their surfaces by adsorbing cells through their porous structures, and the biofilm formation can be judged by FESEM and the change of particle size [23]. Moreover, choosing a material with a density less than that of culture media as a carrier (such as hollow glass microspheres) in the fermentation system can intuitively reflect the biofilm formation process [24].

Biofilm formation is a complex dynamic process, and the attachment of cells to a carrier is one of the most important processes, representing a turning point from planktonic to the biofilm life mode [18]. Cell appendages such as flagella, fimbriae and pili are involved in this stage. The second messenger cAMP, generated by adenylate cyclase CyaA, has been shown to regulate tad IV pili [25]. Tad IV pili are important surface appendages that are central to the surface-sensing mechanism in the early stages of biofilm formation [26]. High type IV pili/cAMP levels might result in a greater attachment tendency [26]. The expression levels of *cyaA* and Bbi37|peg.1398 (cAMP receptor) were high during biofilm formation, and Bbi37|peg.1398 was upregulated during the biofilm growth stage. Bifidobacteria have been shown to encode type IV pili, which are associated with biofilm formation and colonization [27,28,29]. Moreover, sortase-dependent pili identified in *B. bifidum* PRL2010 were shown to promote self-aggregation and aggregation with other gut bacteria [30,31]. Type IV tad pili and quorum sensing are important for the early stages of biofilm formation [26,32].

Interestingly, the functions of DEGs were mainly related to abiotic stimulation, stress response, protein and polysaccharide biosynthesis, two-component system, quorum sensing and global regulatory factors. The genes upregulated were a global phenomenon during *B. bifidum* biofilm formation (Figure 2, Figure 3, Figure 4 and Figure 5): in the early stage of the biofilm, genes related to stress response were upregulated; after the adhesion was completed, cells begin to secrete EPSs leading to biofilm growth, and the related genes were upregulated. YidC, which functions as an integral membrane chaperone/insertase associated with the SecYEG translocon, was identified as a target that can inhibit biofilm formation [33]. Elimination of *yidC* paralogs in *Streptococcus* disrupts EPS composition and biofilm development [34]. The tad IV pili gene (*tadB*) and YidC protein gene (*yidC*) were both upregulated during biofilm formation (Figure 2d). The two-component system, consisting of a histidine kinase and a cognate response regulator [35], plays an important role in monitoring internal or environmental signals and then translating these stimuli into appropriate cellular responses, and it is also associated with bacterial biofilm formation [36]. Notably, four DEGs (Bbi37|peg.1341, *degP*, *pstS*, *vanY*) associated with the two-component system were found during *B. bifidum* biofilm formation (Figure 3). The ∆*degP* probiotic *Escherichia coli* strain exhibited 20-fold lower biofilm formation than the wild-type strain and lost the ability to inhibit pathogenic biofilm formation via a DegP-mediated interaction [37].

The function of differentially expressed metabolites indicates that amino acid, short-chain fatty acids and vitamins were the main metabolites and their expression levels were different during biofilm formation (Figure 7). Amino acids serve as precursors for energy generation with gluconeogenesis, and less abundance of amino acids in the biofilm state of *B. bifidum* reflects less energy production, which indicates the biofilm had already attained maturity [16]. Biofilm formation requires cell adhesion, surface conditioning and EPS production, which are energetically expensive processes; however, it is evolutionary justified given the great benefits for bacteria to live embedded in the EPS matrix under oligotrophic conditions; therefore, nascent biofilms in eutrophic systems have reduced lag phases and higher growth rates than biofilms from oligotrophic systems [3,38,39]. In bacterial biofilms, organic acids, including short-chain fatty acids, are mainly produced by the fermentation of sugars and can reduce the pH value [40]. The rate of biofilm formation by *Actinomyces naeslundii* cells was upregulated by 6.25 m_M_ butyric acid compared to the rate in the control (no short-chain fatty acids) in 96-well microtiter plates, and this upregulation was mediated by GroEL (the heat shock protein) [41]. Another report indicated that the number of biofilms consisting of *A. naeslundii* cells generated from initial attachment cells in a flow cell system was increased by treatment with 60 mM butyric acid [42]. These reports suggested that the effects of short-chain fatty acids, including butyric acid, could induce the potentiation of the cell status required for initial cell attachment, colonization and biofilm formation [42,43]. Ascorbic acid (vitamin C) has previously been reported to enhance oxidative stress tolerance in *Pichia caribbica* [44].

## 4. Materials and Methods

### 4.1. Planktonic and Biofilm Culture

*B. bifidum* FHB150 was obtained from the Jiangnan University (Wuxi, China) and grown anaerobically at 37 °C in MRS broth supplemented with 0.5 g/L L-cysteine hydrochloride monohydrate. Overnight *B. bifidum* FHB150 culture was inoculated (4%, *v*/*v*), and the culture was grown at 37 °C and 120 rpm for 32 h. WF with an average diameter of 50 μm was added to culture media with 4% (*w*/*v*) for biofilm culture [45,46]. No WF-supplemented groups were used as planktonic culture. The CFU and pH values in the WF and control culture group were determined at 10, 22 and 32 h during fermentation; for total cell counting in the WF culture group, samples were vortexed (30 s), sonicated (10 s) and vortexed again (30 s) to disperse biofilm cells into the suspension (Vibra Cell Model VCX150PB, Sonics & Materials Inc., Danbury, CT, USA) [24,47]. 

### 4.2. FESEM

Particles in the WF culture were filtered by a cell sieve (with a diameter of 40 µm), dehydrated with ethanol for 10 min, coated with gold and examined using FESEM (SU8220, Hitachi High-Technologies, Tokyo, Japan) [48]. The low-magnification images acquired by scanning electron microscopy can show the 3D architecture of the biofilm, while the high-magnification images can show the single-cell morphology and EPS organization [16,48].

### 4.3. Transcriptomic Analysis

#### 4.3.1. RNA-Seq and Reads Mapping

RNA-seq was conducted using an Illumina HiSeq 4000, and 8 FASTQ files of sequences were yielded (22 h P, 22 h B, 32 h P, 32 h B; two biological replicates per condition). Transcriptome data of *B. bifidum* FHB150 during biofilm formation have been deposited in the National Center for Biotechnology Information (NCBI) database under BioProject accession code PRJNA733339. The paired-end reads were preprocessed using fastp [49], and reads were aligned to *B. bifidum* FHB150 genomes using HISAT2 v2.20 [50]. Raw read counts were created using featureCounts [51].

#### 4.3.2. Functional and Pathway Analysis of DEGs

Differential expression analysis of *B. bifidum* biofilm and planktonic cells was performed using the DESeq2 [52]. Specified pairwise transcriptome comparisons (22 h B vs. 22 h P; 32 h B vs. 32 h P; 32 h B vs. 22 h B; 32 h P vs. 22 h P) were performed to identify the main DEGs with an absolute value of log2 FC > 1.0 [24,53]. Venn diagram was used to show the comparison and overlap between DEGs in different biofilm formation stages [54]. KEGG pathway and GO analyses were conducted using clusterprofiler (R package) [55] for DEGs obtained from different stages.

#### 4.3.3. PPI Network Analysis

The search tool for retrieval of interacting genes (STRING) (https://string-db.org) (accessed on 20 June 2021) database, which integrates both known and predicted PPI networks, can be applied to predict functional interactions of proteins [56]. To seek potential interactions between DEGs in biofilm formation stages, the STRING tool was employed [57]. Active interaction sources, including experiments, text mining, databases and co-expression; species limited to “*Bifidobacterium bifidum*”; and an interaction score >0.4 were applied to construct the PPI network. Cytoscape software version 3.8.0 was used to visualize the PPI network [58]. 

### 4.4. Metabolomic Analysis

#### 4.4.1. Metabolite Extraction

The culture samples were centrifuged under 12,000 rpm at 4 °C for 10 min, and the 100 µL supernatants were mixed with 800 µL of ice-cold mixture (methanol–acetonitrile–H_2_O = 2:2:1) for 15 min before lyophilization; finally, the dried samples were stored at −80° for LC-MS assay [59].

#### 4.4.2. LC-MS-Based Metabolome Assay

The column temperature was 40 °C with a flow rate of 0.5 mL/min; the injection volume was 5 µL; mobile phases were water (A) and acetonitrile (B) containing each 0.1% (*v*/*v*) of formic acid in positive mode; mobile phases were water with 1 mM ammonium fluoride and 0.1% formic acid (A) and acetonitrile (B) in negative mode; for both modes, the elution gradient (A:B, *v*/*v*) was as follows: 80:20 from 0 to 1 min, 0:100 in 7 min and kept 4 min, and then 80:20 at 11.5 min and kept 2 min [13,59]. Raw data handling was done using Compound Discoverer software. MetaboAnalyst 5.0 (https://www.metaboanalyst.ca) (accessed on 20 June 2021) was used for metabolic pathway and function analysis.

### 4.5. Statistical Analysis

Data were analyzed using the RStudio (v3.5.0) environment (https://www.r-project.org/index.html) (accessed on 20 June 2021). The R package ComplexHeatmap (v2.5.1) (https://jokergoo.github.io/ComplexHeatmap-reference/book/) (accessed on 20 June 2021) [60] was used to process the heat map. The package ggplot2 (v3.3.2) (https://ggplot2.tidyverse.org/reference/) (accessed on 20 June 2021) was used for graphical representation of data. The difference was calculated using t-test and considered statistically significant at *p* < 0.05.

## 5. Conclusions

In conclusion, a time series global transcription and metabolite profiling of *B. bifidum* FHB150 showed transcriptional and metabolic changes when this strain was cultivated under biofilm and planktonic conditions. Using the combined transcriptomic and metabolomic analysis, the present study suggests that *B. bifidum* biofilm formation is associated with quorum sensing; two-component system and amino acid metabolism. However, further studies are needed to confirm this and to elucidate the exact underlying mechanism of *B. bifidum* biofilm formation.

## Data Availability

All raw data for RNA-seq were deposited into NCBI (BioProject accession code PRJNA734093).
